# Supplementary material for: A lethal mitonuclear incompatibility in complex I of natural hybrids
Source: Nature. 2024 Jan 10;626(7997):119–27. doi: 10.1038/s41586-023-06895-8 (PMC10830419; doi:10.1038/s41586-023-06895-8)
Supplement: Supplementary file 2 — Reporting Summary [file 41586_2023_6895_MOESM2_ESM.pdf]

nature portfolio

Corresponding author(s): Benjamin Moran (benmoran@stanford.edu)
Molly Schumer (schumer@stanford.edu)

Last updated by author(s): Nov 2, 2023

Reporting Summary

Nature Portfolio wishes to improve the reproducibility of the work that we publish. This form provides structure for consistency and transparency in reporting. For further information on Nature Portfolio policies, see our Editorial Policies and the Editorial Policy Checklist.

Statistics

For all statistical analyses, confirm that the following items are present in the figure legend, table legend, main text, or Methods section.

n/a	Confirmed	
☐	☒	The exact sample size (*n*) for each experimental group/condition, given as a discrete number and unit of measurement
☐	☒	A statement on whether measurements were taken from distinct samples or whether the same sample was measured repeatedly
☐	☒	The statistical test(s) used AND whether they are one- or two-sided<br>*Only common tests should be described solely by name; describe more complex techniques in the Methods section.*
☐	☒	A description of all covariates tested
☐	☒	A description of any assumptions or corrections, such as tests of normality and adjustment for multiple comparisons
☐	☒	A full description of the statistical parameters including central tendency (e.g. means) or other basic estimates (e.g. regression coefficient) AND variation (e.g. standard deviation) or associated estimates of uncertainty (e.g. confidence intervals)
☐	☒	For null hypothesis testing, the test statistic (e.g. *F*, *t*, *r*) with confidence intervals, effect sizes, degrees of freedom and *P* value noted<br>*Give P values as exact values whenever suitable.*
☐	☒	For Bayesian analysis, information on the choice of priors and Markov chain Monte Carlo settings
☐	☒	For hierarchical and complex designs, identification of the appropriate level for tests and full reporting of outcomes
☒	☐	Estimates of effect sizes (e.g. Cohen's *d*, Pearson's *r*), indicating how they were calculated

Our web collection on statistics for biologists contains articles on many of the points above.

Software and code

Policy information about availability of computer code

Data collection	Simulation data were collected using SLiM v3.3.1, SELAM v0.8 (https://github.com/russcd/SELAM, commit d89bcd244d7e007e457933ae0a6bc2ba0b94db0f) and custom R code (R v4.3.0) to be made available at https://github.com/Schumerlab/mitonuc_DMI. Mass spectrometry data were collected using Skyline v19.1.0.193.
Data analysis	Raw genotype data were converted to local ancestry probabilities using ancestryinfer (https://github.com/Schumerlab/ancestryinfer) and custom scripts available at https://github.com/Schumerlab/Lab_shared_scripts. Bioinformatic analyses were carried out using BLASTn v2.11.0, samtools v1.8, bcftools v1.8, bwa v0.7.17, Picard Tool v1.118, GATK v3.4-46-gbc02625, PAML v4.8a, SIFT web server (https://sift.bii.a-star.edu.sg/www/SIFT_aligned_seqs_submit.html), Clustal Omega v1.2.4, RAxML v8.2.12, pbAA v0.1.2, and the R packages DESeq2 (v1.42.0) and ppcor (v1.0). Protein structural modelling was performed with the RaptorX web server (http://raptorx.uchicago.edu). Raw embryo respirometry data was processed in Loligo Microresp v1.6, and morphometrics images were processed in ImageJ v1.52q. Heart histology slides were measured in QuPath v0.4.3

For manuscripts utilizing custom algorithms or software that are central to the research but not yet described in published literature, software must be made available to editors and reviewers. We strongly encourage code deposition in a community repository (e.g. GitHub). See the Nature Portfolio guidelines for submitting code & software for further information.

Data

Policy information about availability of data

All manuscripts must include a data availability statement. This statement should provide the following information, where applicable:

- Accession codes, unique identifiers, or web links for publicly available datasets
- A description of any restrictions on data availability
- For clinical datasets or third party data, please ensure that the statement adheres to our policy

Raw sequencing reads used in this project are available under SRA Bioprojects PRJNA744894, PRJNA746324, PRJNA610049, PRJNA361133, and PRJNA745218. Mass spectrometry data are available on PRIDE with identifier PXD046217, and other datasets necessary to recreate the results of the publication are available on Dryad (https://doi.org/10.5061/dryad.j3tx95xmx). Templates for Complex I protein structural modeling were accessed from the Protein Data Bank (PDB) with accession numbers 6G2J, 6G72, 5LDW, 5LNK, and 5XTC.

Research involving human participants, their data, or biological material

Policy information about studies with human participants or human data. See also policy information about sex, gender (identity/presentation), and sexual orientation and race, ethnicity and racism.

Reporting on sex and gender	N/A
Reporting on race, ethnicity, or other socially relevant groupings	N/A
Population characteristics	N/A
Recruitment	N/A
Ethics oversight	N/A

Note that full information on the approval of the study protocol must also be provided in the manuscript.

Field-specific reporting

Please select the one below that is the best fit for your research. If you are not sure, read the appropriate sections before making your selection.

☐ Life sciences ☐ Behavioural & social sciences ☒ Ecological, evolutionary & environmental sciences

For a reference copy of the document with all sections, see nature.com/documents/nr-reporting-summary-flat.pdf

Ecological, evolutionary & environmental sciences study design

All studies must disclose on these points even when the disclosure is negative.

Study description	The study tested for evidence of mitonuclear incompatibilities in hybrid fishes by using a combination of lab-born hybrids and natural hybrid populations. Most quantitative analyses were performed in a linear model framework, with genotypes at key loci as independent variables (along with confounding factors such as genome-wide ancestry, brood, age, etc.) and various phenotypes of interest (survival, respiration rates, morphometrics) as dependent variables.
Research sample	Our sample included a total of 1253 lab-born F2 hybrids between Xiphophorus birchmanni and Xiphophorus malinche, as well as 952 natural late-generation X. birchmanni x malinche hybrids from five populations in three rivers. This combination of lab-raised vs. wild hybrids and early- vs. late-generation hybrids from multiple demographic sources allowed the best possible combination of statistical power, precision, natural replication and experimental tractability in inferring the effects of particular genotype combinations. Wild source populations included Aguazarca (N = 126), Calnali Low (N = 359), and Chahuaco Falls (N = 244) all on the Rio Calnali, as well as Acuapa (N = 117) on the Rio Huazalingo, and Tlatemaco (N = 126) on a tributary to the Rio Claro. All pure X. birchmanni and X. malinche individuals used in the study for experimentation and generation of F2 hybrids were collected from Coacuilco, Hidalgo, Mexico, and Chicayotla, Hidalgo, Mexico. Two additional X. malinche for phylogenetics were collected at Tetipanchalco and Tecpaco, Hidalgo, Mexico. Of the F2 hybrids, 943 were drawn from an existing dataset of adults (6+ months old) raised at the CICHAZ field station in Hidalgo, Mexico for genetic mapping (Powell et al. 2021 Current Biology, SRA BioProject PRJNA692059), 75 were juveniles (3-5 months old) born and raised at Stanford University used to track lethality timing and heart histological phenotypes (one of which was euthanized and not included in statistical analyses), and 235 were embryos dissected out of 10 F1 hybrid mothers at Stanford for embryo respirometry assays. For differential expression and allele-specific expression analyses, we used existing RNA-seq data from a prior project (Payne et al. 2022 Molecular Ecology, SRA BioProject PRJNA746324) containing 3 each of male F1 hybrids, X. birchmanni, and X. malinche. We used a total of 16 X. malinche, 16 X. birchmanni, 14 F1 hybrids, and 9 lab-born progeny of Calnali Low hybrids for qPCR, Oroboros respirometry, mitochondrial membrane potential, and mass spectrometry analyses. For embryo stage analyses in wild hybrids, we dissected embryos out of 38 females from the previously mentioned collections, and successfully genotyped a total of 269 embryos included in the manuscript. We also successfully genotyped 8 and 11 broods dissected from 29 X.

	birchmanni and 36 X. malinche wild-caught females, respectively. For analysis of embryo survival outside the womb, we used a total of 20 X. birchmanni fry from two females and 25 F1 fry from four females. In testing Xiphophorus fry sensitivity to Complex I inhibition, we used a total of 39 X. birchmanni fry from three broods, and 11 X. malinche fry from one brood. To construct de novo whole-mtDNA phylogenies, we used 5 X. birchmanni, 3 X. malinche, and 2 X. cortezi from Huichihuayan, San Luis Potosi, Mexico, and the Xiphophorus Stock Center at Texas State University. For mapping of the incompatibility between X. birchmanni and X. cortezi, we used 284 natural X. birchmanni x cortezi hybrids from Santa Cruz, Huextetitla, and Chapulhuacanito, San Luis Potosi, Mexico.
Sampling strategy	Sample sizes for the Powell et al. F2 cross were determined using a power analysis for a previous QTL mapping study, where sample size was chosen for an estimated 90% chance of detecting a QTL explaining 5% of phenotypic variance. Sample sizes for all individuals collected from natural populations were determined by sampling success during field seasons and the maximum permitted across all sampling sites by collection permits. Sample sizes for all analyses based on lab-born hybrids were either the total number available with the desired genotypes at the time of experimentation, or the maximum feasible while still preserving the viability of lab colonies (which can only periodically be refreshed with wild collections).
Data collection	Data collection procedures are listed in the main text and Supplement (being too extensive to list here). SMB, AED, and JJB performed wet lab work for genotyping, TRG and MS performed bioinformatics for genotyping, and BMM and QKL contributed to both. TRG performed Complex I pharmacological inhibition trials and all other measurements of fry survival. BMM, DLP, and SMB collected embryo stage data from wild-caught hybrids. BMM performed respirometry and morphometrics in live embryos, while ENKI and JCH performed Oroboros respirometry assays on isolated mitochondria. SMB performed qPCR for mitochondrial copy number, CYP performed differential expression and allele-specific expression analyses, and AM performed assays of mitochondrial membrane polarization. FL, RM, KS, and RDL performed mass spectrometry assays on mitochondrial isolates prepared by BMM and AED. RRS analyzed histology images of juvenile F2 hearts. BMM constructed all phylogenies and associated testing of evolutionary rates, performed structural modeling, and carried out coevolution tests. MS and BMM performed all simulation and statistical analyses.
Timing and spatial scale	All natural hybrid samples and parental species were collected over three years of field trips to the CICHAZ field station in Calnali, Hidalgo, Mexico, from ten different sites within an area of Hidalgo and San Luis Potosi ~40 km in diameter. Sampling was roughly quarterly with the exception of the year 2020, when sampling was limited by the COVID-19 pandemic.
Data exclusions	18 individuals were excluded from whole embryo respirometry and morphometrics due to damage (punctured yolks, torn tissue, severe internal bleeding, etc) at the time of photography as identified by BMM; these damages could affect body measurements, and it was unclear when the damage occurred, such that a confounding effect on respirometry measurements could not be ruled out. Likewise, four heart compartment slides were excluded from histology analysis due to visible tearing of the tissue on the slide, which was deemed sufficient to affect cross-sectional area by BMM. These criteria were not pre-established, but the observer was unaware of the experimental treatment group when making the decision to exclude.
Reproducibility	Replicability was largely confirmed by comparing across biologically independent datasets and statistical approaches: the depletion of mismatched genotypes was repeated in segregation distortion in lab-born F2 hybrids, partial correlation analysis in two natural hybrid populations with both mitochondrial ancestries segregating, and permutation-based testing of depletion in three natural hybrid populations fixed for mitochondrial ancestry. Replicability of in silico protein modelling was tested by repeating structure prediction with five different random seeds and four different mammalian cryo-EM templates. Whole embryo respirometry was replicated across ten broods in two major sets separated by ~6 months.
Randomization	Randomization is not relevant to our study because all analyses either lacked experimental treatments or applied a single treatment to all individuals, with the genotypes of individual being the independent variable of interest. Where applicable, the covariate of genome-wide ancestry fraction was controlled for using partial correlation analysis, and any variation in the administration of respirometry methods were accounted for for by using date of testing as a blocking variable in downstream statistics.
Blinding	Blinding was not employed in this study. Many analyses featured only a single group, such that blinding was not relevant, and in others, the placement of individuals in experimental groups of interest (e.g. genotypes) was impossible until after all data collection and analysis had already occurred. In the case of Oroboros respirometry, treatment groups of individuals were visible to experimenters based on phenotype.

Did the study involve field work? ☒ Yes ☐ No

Field work, collection and transport

Field conditions	Collections were performed in September, November, January, February, March, May, and June in the Sierra y Huasteca region of Mexico, with sites ranging from ~10-30 C depending on the season, and rainfall varying from absent during the peak dry season (winter and spring) to frequent and substantial in the rainy season (summer and fall).
Location	Sampling was carried out in 0-2 meter of water at all sites, including:<br>Coacuilco: 21.097544° latitude, -98.588917° longitude, 315 m elevation<br>Chicayotla: 20.924232°, -98.576144°, 1020 m<br>Xontla Falls Up: 20.926958°, -98.588595°, 1195m<br>Tetipanchalco: 20.879285°, -98.799525°, 680 m<br>Aguazarca: 20.898505°, -98.602150°, 980 m<br>Calnali Low: 20.899356°, -98.575438°, 920 m<br>Chahuaco Falls: 20.906892°, -98.537260°, 610 m<br>Tlatemaco: 21.022704°, - 98.790106°, 375 m<br>Acuapa: 20.955922°, -98.571274°, 510 m<br>Santa Cruz: 21.157675°, -98.520497°, 145 m

	Huextetitla: 21.162172°, -98.557554°, 165 m<br>Chapulhuacanito: 21.210835°, -98.670220°, 145 m
Access & import/export	Samples were collected under Collection Permit No. PPF/DGOPA-002/19 issued from the Mexican National Commission of Aquaculture and Fisheries (CONAPESCA, issued 8/28/2020) and imported under Long-Term Importation Permit #2023 - 7129 from the California Department of Fish and Game (issued 2/9/2023).
Disturbance	No disturbance was caused by the study except the removal of Xiphophorus from the streams. Sample sizes were small relative to the population sizes of these species and the timespan over which the samples were collected, but we nonetheless monitored for signs of population perturbance caused by sampling (changes in age distribution, sex ratio, catch per trap, etc.)

Reporting for specific materials, systems and methods

We require information from authors about some types of materials, experimental systems and methods used in many studies. Here, indicate whether each material, system or method listed is relevant to your study. If you are not sure if a list item applies to your research, read the appropriate section before selecting a response.

Materials & experimental systems

n/a	Involved in the study
☒	☐ Antibodies
☒	☐ Eukaryotic cell lines
☒	☐ Palaeontology and archaeology
☐	☒ Animals and other organisms
☒	☐ Clinical data
☒	☐ Dual use research of concern
☒	☐ Plants

Methods

n/a	Involved in the study
☒	☐ ChIP-seq
☒	☐ Flow cytometry
☒	☐ MRI-based neuroimaging

Animals and other research organisms

Policy information about studies involving animals; ARRIVE guidelines recommended for reporting animal research, and Sex and Gender in Research

Laboratory animals	Laboratory animals included adult male and female Xiphophorus birchmanni and X. malinche, as well as F1 and F2 hybrids between them. Xiphophorus fry were fed newly hatched (<24 hours old) brine shrimp (Artemia franciscana, Great Salt Lake strain)
Wild animals	Wild-caught animals used in this study included male and female X. birchmanni, X. malinche, X. pygmaeus, X. cortezi, X. birchmanni x malinche hybrids, and X. birchmanni x cortezi hybrids. X. pygmaeus and X. cortezi individuals were all adult (sexually mature, exact ages are impossible to know in wild fish but likely greater than 6 months post-birth), while all other sampling included embryos (less than ~30 days post-fertilization and pre-birth), juveniles (sexually immature, likely <6 months post-birth), and adults (sexually mature, likely >6 months post-birth). Individuals were caught with baited minnow traps and transported in minnow buckets for local travel and methylene-blue treated water containers for long-distance travel. If lethal sampling was not necessary, captive animals were either non-lethally fin clipped for DNA before release within a week of capture at the point of capture, or returned to the laboratory to establish breeding colonies. Dissection was necessary to remove embryos or liver for RNA, protein, and respirometry analyses; in these cases, captive animals were euthanized using lethal overdoes of tricaine (MS-222).
Reporting on sex	Sex was not considered in analyses involving embryos, fry, or juveniles, as sexual differentiation had not yet occurred and the sex determining loci for X. birchmanni and X. malinche have yet to be identified. Sex was balanced across groups and tested for significant effects in the Oroboros mitochondrial respiration assays, but had no significant effect on any parameters. Analyses based on genotypes of wild-caught individuals include males and females in the proportions captured, and so selection and dominance coefficients referenced in the manuscript are sex-averaged. RNA-seq and membrane polarization assays were performed exclusively on males, and mass spectrometry exclusively on females, to exclude potential sex effects.
Field-collected samples	This study did not involve samples collected from the field
Ethics oversight	Ethical approval was provided by the Stanford Administrative Panel on Laboratory Animal Care (APLAC), protocol #33071

Note that full information on the approval of the study protocol must also be provided in the manuscript.
